# Dissatisfaction-considered waiting time prediction for outpatients with interpretable machine learning

**DOI:** 10.1007/s10729-024-09676-5

**Published:** 2024-06-01

**Authors:** Jongkyung Shin, Donggi Augustine Lee, Juram Kim, Chiehyeon Lim, Byung-Kwan Choi

**Affiliations:** 1 Graduate School of Artificial Intelligence, Ulsan National Institute of Science and Technology, 50 Unist-gil, Eonyang-eup, Ulju-gun, 44919 Ulsan, Republic of Korea; 2 Microsoft Technology Centers, Microsoft Korea, 50, Jongno 1-gil, Jongno-gu, 03142 Seoul, Republic of Korea; 3 Center for R &D Investment and Strategy Research, Korea Institute of Science and Technology Information, 66 Hoegi-ro, Dongdaemun-gu, 02456 Seoul, Republic of Korea; 4 Department of Neurosurgery, Pusan National University Hospital, 179, Gudeok-ro, Seo-gu, 49241 Busan, Republic of Korea

**Keywords:** Outpatient service, Patient dissatisfaction, Waiting time prediction, Asymmetric loss function, Interpretable machine learning

## Abstract

Long waiting time in outpatient departments is a crucial factor in patient dissatisfaction. We aim to analytically interpret the waiting times predicted by machine learning models and provide patients with an explanation of the expected waiting time. Here, underestimating waiting times can cause patient dissatisfaction, so preventing this in predictive models is necessary. To address this issue, we propose a framework considering dissatisfaction for estimating the waiting time in an outpatient department. In our framework, we leverage asymmetric loss functions to ensure robustness against underestimation. We also propose a dissatisfaction-aware asymmetric error score (DAES) to determine an appropriate model by considering the trade-off between underestimation and accuracy. Finally, Shapley additive explanation (SHAP) is applied to interpret the relationship trained by the model, enabling decision makers to use this information for improving outpatient service operations. We apply our framework in the endocrinology metabolism department and neurosurgery department in one of the largest hospitals in South Korea. The use of asymmetric functions prevents underestimation in the model, and with the proposed DAES, we can strike a balance in selecting the best model. By using SHAP, we can analytically interpret the waiting time in outpatient service (e.g., the length of the queue affects the waiting time the most) and provide explanations about the expected waiting time to patients. The proposed framework aids in improving operations, considering practical application in hospitals for real-time patient notification and minimizing patient dissatisfaction. Given the significance of managing hospital operations from the perspective of patients, this work is expected to contribute to operations improvement in health service practices.

## Highlights

- Proposing an analytical framework for estimating waiting times in outpatient departments.
- Applying Shapley additive explanation (SHAP) to interpret the relationship between waiting times and operational features.
- Leveraging asymmetric loss functions to prevent underestimation of waiting times in the framework.
- Introducing a dissatisfaction-aware asymmetric error score (DAES) to balance the trade-off between underestimation and accuracy.
- Demonstrating the framework’s effectiveness through a case study in a hospital’s endocrinology metabolism department and neurosurgery department.

## Introduction

Due to the large demand for outpatient services, overcrowding in outpatient departments is common in hospitals (e.g., in the United States [1] and Asian countries [2]). The long waiting times resulting from overcrowding is the main cause of patient dissatisfaction, which causes a decline in perceived service quality [3, 4]. Therefore, managing waiting times is necessary to improve the quality of outpatient services, and it is an issue that has been considered extremely important for hospital operations management [5, 6].

Given this need, previous studies have attempted to measure the importance of service features affecting outpatient waiting times using linear models (e.g., linear regression (LR)) that statistically estimate the extent to which different features affect service quality based on questionnaire data collected from outpatients [7–9]. However, this approach has at least two limitations. First, the traditional technique can represent the intuitive relationship between waiting times and considered features. However, it exhibits low predictive accuracy because linear assumptions may not be suitable to accommodate the complex patterns in hospital operation systems [10, 11]. Second, measurement using outpatient questionnaires is expensive and time-consuming [8]. Moreover, this approach mainly represents outpatient perspectives on isolated temporal snapshots, whereas actual service improvement requires the continuous analysis of service operations to allow for real-time inferences [12].

To address these limitations, technologies using data collected from hospital information systems (HIS) and machine learning (ML) have been studied to improve hospital services [13–16]. As a result, waiting time management has benefited substantially from the use of prediction models trained with rich data to estimate outpatient waiting times [10, 17, 18]. However, although this data-driven ML approach achieves high accuracy, the inability to interpret the predictions makes it challenging to estimate the importance of service features that affect waiting times. To address the lack of interpretability of ML models, several studies have suggested and employed interpretable machine learning (IML) approaches in healthcare. For instance, Ahmad et al. [19] and Stiglic et al. [20] emphasized the significance of IML and its applicability to address challenges and requirements within healthcare. Gao et al. [21] utilized a two-step extracted regression tree approach for hospital readmission prediction, achieving a balance between accuracy with interpretability. Okay et al. [22] applied IML methods in conjunction with random forest (RF) and gradient boosting algorithms to diagnose diabetes, enhancing interpretability without sacrificing accuracy. Finally, Hu et al. [23] employed IML to understand the reasoning behind the outcome of an optimal model for the early prediction of prognosis in sepsis. However, specific efforts to apply IML for predicting patient waiting times in healthcare are still lacking despite the importance of the prediction model’s interpretability from an operational perspective. Without an analytical interpretation of the relationship between service features and waiting times, hospital managers face difficulty in prioritizing which features to adjust to reduce waiting times and enhance overall patient satisfaction.

Meanwhile, given that outpatients are notified of the expected waiting time, a significant research gap in the literature is the lack of ML models that reflect patients’ perspective in the training phase. Providing patients with individual expected waiting times improves patient satisfaction, as it enables them to manage their time effectively instead of passively waiting for their consultation in the waiting room. From the hospital’s perspective, a prediction model can be useful for allocating workloads to medical workers through simulation analysis, such that operations can be improved to alleviate patient dissatisfaction. However, this plausible scenario considers an important assumption, that is, the model accurately predicts the waiting time for each patient. In case of overestimation, the actual waiting time being less than the predicted waiting time can be acceptable and may even have a positive effect on patient satisfaction [24]. However, a serious concern of hospital staff regarding the introduction of predictive models is the underestimation by the models. If a patient cannot enter the consultation room even after the notified waiting time, then dissatisfaction may increase with the patients’ perceived waiting time  [25]. This situation occurs frequently in practice. In addition, if the perceived waiting times based on the patient’s situational observation of the waiting room and the predicted waiting times are different, then patient dissatisfaction can increase [26, 27]. In summary, to reduce patient dissatisfaction when introducing a prediction model for waiting time notification, it is important that the ML model does not underestimate patients’ waiting times. Additionally, it is crucial to identify and explain the specific reasons for long waiting times to the patients.

**Fig. 1:**
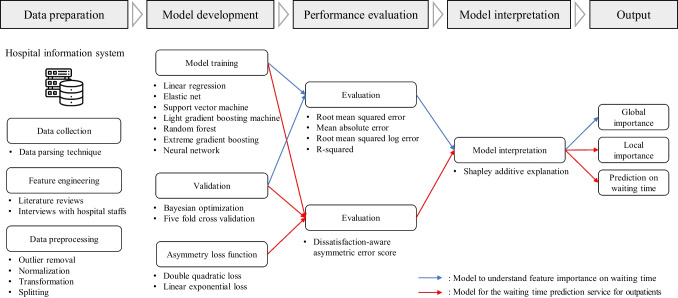
Overall process of the proposed approach

To address these issues, we propose a framework for waiting time prediction considering patient dissatisfaction using the IML method and asymmetric loss functions. First, IML can explain the mechanisms of ML models trained by black-box models [28], thereby addressing the trade-off between predictability and interpretability. Second, the loss functions employed in existing studies to train ML models are symmetric, which compute equivalent penalties that may result in overestimation and underestimation. By contrast, the asymmetric loss functions used in our study calculate different penalties depending on either the sign of the error value or the specific interval it contains; even the absolute value is equivalent. Third, given that the introduction of an asymmetric loss function adversely affects the accuracy of ML models, the dissatisfaction-aware asymmetric error score (DAES) introduced in this study allows for the selection of a suitable model by considering the trade-off between accuracy and underestimation. Hence, our framework allows the accurate prediction of outpatient waiting times and estimation of the importance of service features affecting the waiting times from a service-oriented perspective. The objective is to reduce patients’ dissatisfaction caused by the underestimation of waiting times. Furthermore, our framework can alleviate the underestimation problem of the prediction model by the asymmetric loss function and provide an explanation of the reason for each waiting time prediction. Finally, with our framework, decision makers can interpret the prediction model to identify directions for improving outpatient service operations.

We validate the proposed framework through case studies in one of the largest hospitals in South Korea. We analyze continuously collected operations data archived in the HIS and define the variables to construct datasets for developing prediction models. Then, we investigate the importance of features on waiting time prediction and discuss the theoretical and practical implications of the analysis results in this paper. The results of the case study confirmed that the proposed analytical framework is a useful tool for waiting time management, and serves as a starting point for gaining a deeper understanding of the relationships between the factors in outpatient service and waiting time. Furthermore, the model trained with asymmetric loss functions considering patient dissatisfaction can be implemented in a system providing stakeholders with the expected waiting time and the corresponding explanation to enhance patient satisfaction and reduce the perceived waiting time. In practice, outpatient waiting time prediction should be used for patient notification and improvement in outpatient operations. In conclusion, our framework goes beyond mere prediction; it offers practical solutions to the challenges faced in outpatient healthcare settings. By providing real-time patient notifications, enabling operations improvements, and minimizing patient dissatisfaction, our work contributes significantly to the field, ultimately benefiting both patients and healthcare providers.

To further illustrate the contribution of our work, the remainder of this paper is organized as follows. Section 2 describes the overall framework and methodological background, including Shapley additive explanations (SHAP), the asymmetric loss function, and DAES. Section 3 describes the results of applying the proposed framework to an actual hospital in South Korea. Finally, the conclusions of this study are presented in Section 4.

## Methodology

### Proposed framework

The overall process of the proposed approach is shown in Fig. 1 and comprises four steps: (1) data preparation, (2) model development, (3) performance evaluation, and (4) model interpretation. The blue line in the figure represents the process for understanding the features that affect waiting times for service improvement, and the red line represents the process for developing a model for waiting time prediction service. The data preparation step involves data collection from the HIS, feature engineering through literature review and interviews, and data preprocessing. In model development and performance evaluation steps, ML models are trained to accurately estimate the waiting time by hyperparameter optimization, and the performance of the trained ML models is examined by evaluation metrics to determine the best model. Finally, SHAP (Section 2.2) is applied to perform an analytical interpretation of the relationship between waiting times and the considered features. For the waiting time prediction service, the ML models are trained by asymmetric loss function (Section 2.3) and evaluated by DAES (Section 2.4) to consider patient dissatisfaction.

### SHAP

SHAP is a model-agnostic IML method based on coalition game theory; it calculates the Shapley value representing the contribution of each player [29, 30]. SHAP has been shown to satisfy the properties of local accuracy, consistency, and missingness [29]. Among various IML methods, such as Local Interpretable Model-agnostic Explanations [31] and Anchors [32], we employ the SHAP for the following reasons. First, SHAP is based on the rigorous theoretical background of Shapley values used in game theory [30] and offers explanations of the model’s results without sacrificing its accuracy, ensuring a solid scientific basis for our interpretability framework. Second, SHAP allows for sample-wise interpretability, offering customer-centric insights tailored to individual customers. Each patient can be provided with an explanation of the expected waiting time, increasing their satisfaction and awareness of the waiting situation. Third, from a healthcare operations perspective, SHAP helps us understand global feature importance and analyze the significance of specific data subsets, facilitating optimized resource allocation and operational efficiency. Finally, this method is model-agnostic and universally applicable without the need for custom parameter tuning. It excels in considering complex interplays between variables, making it valuable for our analysis of factors affecting outpatient service waiting times in hospitals. Moreover, SHAP assigns importance to each feature through additive feature attribution, which is calculated as follows:

1 $$\begin{aligned} g(z') = \varphi _{0} + \sum ^{M}_{i=1} \varphi _{i}z'_{i} \end{aligned}$$

where *g* is the explanation model; $z'\in \{0,1\}^{M}$ is the coalition vector that represents whether the feature is present (=1) or absent (=0); *M* is the number of features; $\varphi _{0}$ is the intercept value; and $\varphi _{i} \in \mathbb {R}$ is the feature attribution for feature *i*, the SHAP value.

Specifically, the SHAP value of feature *i* is calculated using the expected marginal contribution. This is the difference between the importance value without feature *i* and the importance of the entire subset, as shown in Eq. (2).

2 $$\begin{aligned} \small \varphi _{i} = \sum _{S \subseteq S_{all} \backslash \{ i \}} { \vert S\vert !(M-\vert S \vert -1)! \over M! } (f_{x} (S \cup \{ i \} - f_{x}(S)) \end{aligned}$$

where $\varphi _{i}$ denotes the SHAP value of feature *i*, *S* denotes the subset of features, and $S_{all}$ denotes the set of all features. $f_{x}(S \cup \{ i \})$ and $f_{x}(S)$ are the conditional expectations of model *f* with and without feature *i*, respectively, at an observed variable *x* belonging to *S*.

The SHAP value is useful in determining the local importance of individual instances. However, calculating the average absolute SHAP value across all instances enables us to determine the global importance, as shown as follows:

3 $$\begin{aligned} GI_{i} = {1 \over N} \sum ^{N}_{j=1} \vert \varphi _{i}^{(j)} \vert \end{aligned}$$

where $GI_{i}$ is the global importance of feature *i*, *N* is the number of data instances, and $\varphi _{i}^{(j)}$ is the SHAP value of feature *i* for the $j^{th}$ instance.

To reduce time complexity, SHAP values can be approximated by various methods, such as kernelSHAP (applicable to all ML models), DeepSHAP (applicable to neural network models), and TreeSHAP (applicable to decision-tree-based ensemble learning models) [29, 33].

**Fig. 2:**
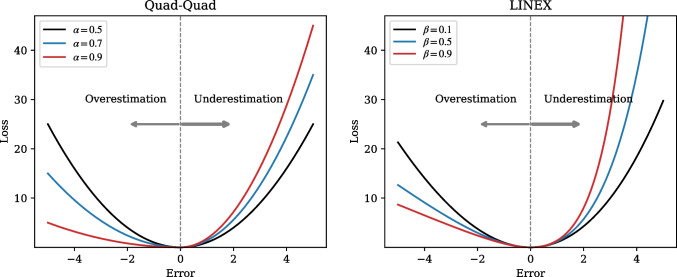
Examples of Quad-Quad and LINEX

### Asymmetric loss function

As aforementioned in the Introduction section, avoiding underestimation is crucial in practical applications, in addition to predictive ability. The reason is that underestimation is more critical to patient dissatisfaction than overestimation. Therefore, we introduce the asymmetric loss function to train the waiting time predictive model. Generally, loss functions, such as mean square errors (MSE), are symmetric and derive the same penalty regardless of the sign of the error value. Different from symmetric loss functions, asymmetric loss functions can derive different penalties depending on overestimation or underestimation. In our study, we employ two popular asymmetric loss functions. These functions contain a quadratic or exponential component that makes them smooth and differentiable, thereby allowing the model to be optimized using gradient-based methods. Figure 2 illustrates the shapes of these loss functions when the parameters are set to penalize underestimation more heavily. This adjustment allows a predictive model to be robust against underestimation by using the asymmetric loss function.

The double quadratic loss function (Quad-Quad) increases quadratically on each side of the origin, but its penalty differs depending on the sign of the error. According to [34], Quad-Quad can be formulated as follow:

4 $$\begin{aligned} L_{quad}(\alpha ) = 2 \cdot [\alpha + (1 - 2\alpha ) \cdot 1_{\{\epsilon <0\}}] \cdot {|\epsilon |}^{2} \end{aligned}$$

where $\epsilon = y - \hat{y}$ is the error between the target value (*y*) and prediction ($\hat{y}$). $1(\cdot )$ is a unit indicator equal to 1 if $\epsilon < 0$, and 0 otherwise. $\alpha \in (0,1)$ is a parameter that adjusts the degree of asymmetry. When $\alpha$ is 0.5, the error is symmetric. If $\alpha > 0.5$, the penalty for underestimation (i.e., $\epsilon > 0$) is larger than that for overestimation (i.e., $\epsilon < 0$).

The linear exponential loss function (LINEX) [35] increases linearly on one side of the origin and exponentially on the other side. This function is convex and can handle asymmetry smoo-thly [36, 37]. The function of LINEX is defined as follows:

5 $$\begin{aligned} L_{linex}(\beta ) = {2 \over {\beta }^{2}} [\exp ({\beta \epsilon }) - \beta \epsilon -1] \end{aligned}$$

where $\beta \ne 0$ is a parameter that adjusts the asymmetry degree, and $\exp (\cdot )$ denotes the exponential function. When $\beta > 0$, positive errors incur more penalties than negative errors.

### Asymmetric error score for dissatisfaction-aware waiting time prediction

We leverage asymmetric loss functions (i.e., Quad-Quad and LINEX), such that the prediction model encourages avoiding underestimation. The model trained by the asymmetric loss function suitably increases the overall prediction values; however, it causes excessive errors in the overestimated predictions. This leads to another dissatisfaction issue when the patients’ turn for consultation is missed, especially for patients who believe in an excessively overestimated result and leave the waiting room. To address this issue, we propose DAES to determine an appropriate deployment model. It provides high penalties in the case of not only underestimated errors but also overestimated errors that satisfy certain conditions. Therefore, the model with the lowest DAES is the most suitable. DAES is formulated as follows:

6 $$\begin{aligned} DAES(\gamma ) = \rho _{(\epsilon> 0)} \cdot e(\epsilon > 0) + \rho _{(\epsilon<-\gamma )} \cdot e(\epsilon <-\gamma ) \end{aligned}$$

where $\epsilon >0$ and $\epsilon <-\gamma$ represent the underestimated and overestimated errors larger than $\gamma$ min, respectively; $\rho$ denotes the ratio of each condition; and $e(\cdot )$ denotes a performance metric, such as the root mean square error (RMSE).

**Fig. 3:**
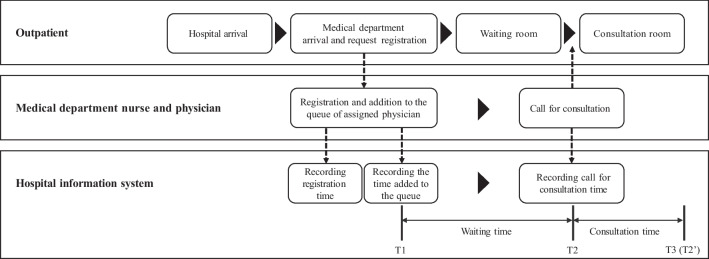
Outpatient visiting process and operational process in the hospital

**Table 1:** Example of the operational event log dataset

Index	Patient ID (Anonymous)	Assign physician	Sequence	Activity	Event Time
1	Patient A	Physician A	1	Registration	2020-08-03 10:15:21
2	Patient A	Physician A	2	Addition into queue	2020-08-03 10:15:22
3	Patient B	Physician A	1	Registration	2020-08-03 10:15:36
4	Patient B	Physician A	2	Addition into queue	2020-08-03 10:15:38
5	Patient C	Physician B	1	Registration	2020-08-03 10:33:20
6	Patient C	Physician B	2	Preliminary examination	2020-08-03 10:33:26
7	Patient A	Physician A	3	Call for a consultation	2020-08-03 10:33:28
8	Patient B	Physician A	3	Call for a consultation	2020-08-03 10:36:12
9	Patient C	Physician B	3	Addition into queue	2020-08-03 11:23:45
...	...	...	...	...	...

## Application results

In this section, we present case studies of an endocrinology metabolism (EM) department and a neurosurgery (NS) department in one of the largest hospitals in South Korea. Our target hospital is located in a metropolitan city with more than 3 million residents. In addition, more than 2 million people living near cities visit this hospital. As a result, approximately 900,000 outpatients visit this hospital annually. We initially applied our framework to the EM department to illustrate the use of the proposed approach. The EM department is one of the most crowded departments in this hospital with approximately 200 patients visiting each day. Patients experience a relatively long waiting time for consultation at an average of approximately 40 min, which has been identified as the main cause of dissatisfaction with EM outpatient services. Therefore, we applied our framework to develop a satisfaction-oriented and accurate waiting time prediction model and analyzed the effects of various features on waiting time.

**Table 2:** Description of features

Factor	Variable	Description	Data type
Queue	Length of queues ($Q_L$)	Number of outpatients assigned to the doctor’s queue for consultation	Continuous
	Return outpatient ratio ($Q_R$)	Percentage of patients visiting within a year	Continuous
	Newly visiting outpatient ratio ($Q_N$)	Percentage of patients visiting for the first time	Continuous
	First-time outpatient ratio ($Q_F$)	Percentage of patients who did not visit in more than a year	Continuous
	Department first-time outpatient ratio ($Q_D$)	Percentage of patients who visited other departments but are visiting this department for the first time	Continuous
Patient	Appointment status ($A_S$)	Whether the patient made an appointment	Categorical
Time	Smooth flow time zone ($T_S$)	Whether the patient registration time within the periods when patient flow proceed efficiently	Categorical
Physician	Average consultation time within $\tau$ min ($P_{ACT}$)	The average consultation time of a doctor within $\tau$ min prior to the patient’s arrival	Continuous
	Number of patients within $\tau$ min ($P_{NP}$)	The number of patients consulted by the doctor within $\tau$ min prior to the patient’s arrival	Continuous

### Data collection

As medical workers (e.g., nurses and physicians) provide services to patients, operational data such as the status of patients and log histories of workers are automatically recorded in the HIS. Specifically, the HIS of the EM department in our target hospital includes a text-to-speech-based electronic calling system that allows workers to call patients into the consultation room. Thus, related operational event log records (e.g., call times and call events) were collected.

Figure 3 shows the outpatient process and corresponding operational processes recorded in the HIS of the EM department. When a patient at the front desk requests to register for medical consultation after arriving at the hospital, a nurse collects the patient’s personal information and checks his/her status. According to the patient’s appointment status and necessity of any preliminary examination, the nurse determines when to add the patient to the queue after registration. Typically, most patients are queued immediately after registration; however, patients who require a preliminary examination are queued after an examination. Therefore, we define the start of waiting (T1) as the moment at which patients were queued and recorded in the HIS. The moment when a physician calls the patient for consultation is regarded as the start of consultation (T2). Moreover, the moment when the physician calls the next patient after completing the consultation is regarded as the end of the consultation (T3) and the start of the consultation of the next patient (T2’). Table 1 presents a dummy dataset for describing the operational event log data stored in the HIS server.

Waiting for outpatient services occurs before consultation; thus, the waiting time is defined as the time difference between T2 and T1. Consultation time is the period in which the patient consults with a physician; hence, it is defined as the time difference between T3 and T2. For this research, we extracted the sample operational event log data from 06/01/2020 to 09/30/2020 for 7,709 outpatients who visited the EM department in our target hospital. Data collection for this period was determined by the hospital with the Institutional Review Board (IRB) approval only for research purposes, and the data did not include patient identification information.

### Feature engineering

Through literature reviews and discussions with nurses and physicians, we categorized four factors that represent the context of waiting and affect patients’ waiting times. The factors influencing the waiting times were queue, patient, time, and physician [7, 18, 38, 39]. Moreover, we defined the features that present the properties of these factors, which could be used to provide an explanation to the patients. Particularly, the features were measured only with collectible information when the patient came to the front desk. Table 2 introduces the factors, their corresponding feature labels, and their descriptions. A detailed description of the feature engineering process is provided in the following subsection. The explanation and interpretability of the model depend on input features. Thus, the features must be carefully selected to be suitable and relevant, such that medical workers can be convinced of the interpretation results, and patients can understand the explanations given by the workers. In view of this issue, we determined the features after interviews with medical workers and observations of patient flow in outpatient services.

#### Characteristics of the queue

We defined the features representing the characteristics of the queue as the length of the queue and proportion of each patient type in the queue. The length of the queue has generally been considered a queue feature that is most relevant for waiting time prediction [7, 10, 40, 41]. Moreover, according to queuing theory [38], the average waiting time is positively correlated with queue length; thus, queue length is regarded as a necessary feature. We confirmed through discussions with nurses that patient type could affect the consultation time. For example, a returning patient often has a relatively short consultation time compared with a newly visiting patient. However, a patient’s waiting time could also be affected by the sum of the consultation times of the patients in the queue of the physician to whom they were assigned. Therefore, the proportions of each patient type in the queue were added as features related to the queue.

#### Characteristics of the patient

Patient characteristics influenced the order in which the nurses assigned patients to the outpatient care service of our target hospital. The nurses at the hospital had patients with appointments preferentially queued ahead of patients without an appointment. In other words, patients who made appointments were placed in the queue immediately after registration, whereas patients without an appointment were delayed in entering the queue. These delays could lead to an increase in waiting time. Therefore, whether a patient had an appointment was selected as a feature to represent the patient’s characteristics.

**Fig. 4:**
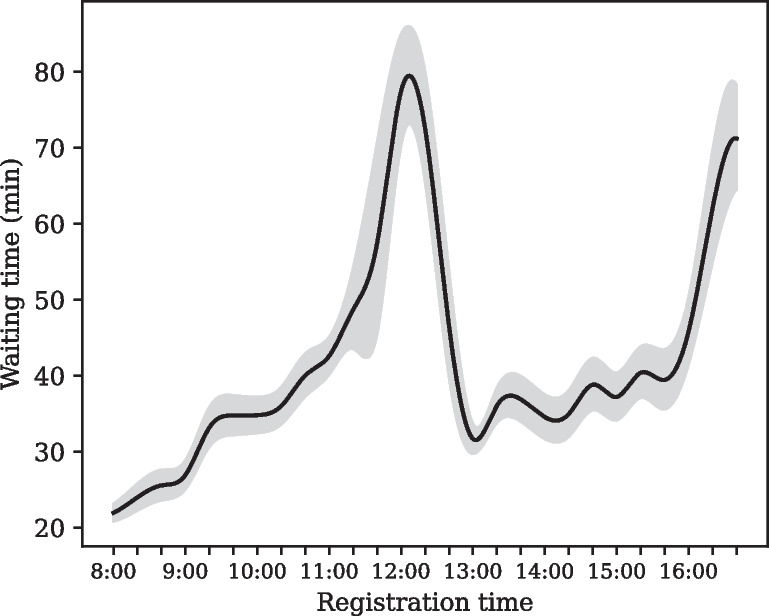
Trend in waiting time distribution by registration time

**Table 3:** Descriptive statistics of the dataset

	Whole dataset (outliers included)	Training dataset (outliers excluded)	Test dataset (outliers excluded)
**Number of instances**	11,563	8,970	2,250
**Waiting time in minutes**			
Min, Max	0.067, 283.617	0.10, 125.733	0.10, 124.017
Mean (SD)	37.482 (25.144)	36.794 (23.717)	36.619 (23.001)
**Consultation time in minutes**			
Min, Max	0.033, 15.00	0.033, 15.00	0.100, 14.98
Mean (SD)	4.730 (2.698)	4.715 (2.687)	4.779 (2.750)
**Length of queues**			
Min, Max	0, 27	0, 27	0, 24
Mean (SD)	5.859 (4.181)	5.824 (4.133)	5.863 (4.082)
**Return outpatient ratio**			
Min, Max	0, 1	0, 1	0, 1
Mean (SD)	0.860 (0.267)	0.859 (0.268)	0.861 (0.261)
**Newly visiting outpatient ratio**			
Min, Max	0, 1	0, 1	0, 1
Mean (SD)	0.012 (0.059)	0.013 (0.061)	0.012 (0.056)
**First-time outpatient ratio**			
Min, Max	0, 1	0, 1	0, 1
Mean (SD)	0.030 (0.090)	0.030 (0.091)	0.030 (0.088)
**Department first-time outpatient ratio**			
Min, Max	0, 1	0, 1	0, 1
Mean (SD)	0.043 (0.129)	0.043 (0.130)	0.044 (0.127)
**Appointment status (Count)**			
Yes (%)	10,509 (90.9%)	8,152 (90.9%)	2,034 (90.4%)
No (%)	1,054 (9.1%)	818 (9.1%)	216 (9.6%)
**Smooth flow time zone (Count)**			
Yes (%)	4,839 (41.8%)	3,749 (41.8%)	943 (41.9%)
No (%)	6,724 (58.2%)	5,221 (58.2%)	1,307 (58.1%)
**Average consultation time within 60 min**			
Min, Max	0, 14.90	0, 14.90	0, 13.717
Mean (SD)	4.812 (1.578)	4.834 (1.572)	4.767 (1.507)
**Number of patients within 60 min**			
Min, Max	0, 19	0, 19	0, 18
Mean (SD)	6.639 (4.495)	6.660 (4.508)	6.582 (4.518)

#### Characteristics of time

Our target hospital operates the outpatient service from 09:00 to 17:30, excluding lunchtime from 12:00 to 13:00. The service is organized into morning and afternoon consultations, with registration opening at 08:00. Several observations of the service flow revealed that the front desk tended to become busier over time. Specifically, patient intake showed smooth flow when each consultation began, that is, in the early morning (between 08:00 and 10:00) and right after lunchtime (between 13:00 and 15:00). However, at the end of each consultation period, patient intake intensified and became the most congested. This tendency resulted in oversaturation of work for the nurses because it required them to process patients’ registration immediately. Such an abnormal process at certain times could cause a delay in nurses’ work, such as registration and addition to the queue for patients, which could result in an increase in patient waiting time. Thus, time zone features were utilized as proxy variables for this phenomenon. Figure 4 illustrates a trend in the distribution of waiting time by registration time^1^. The waiting time is nonstationary, demonstrating its dependence on the registration time, which aligns with our observations. As a result, we set the period between 08:00 and 10:00 and between 13:00 and 15:00 as the smooth flow time zone.

#### Characteristics of the physician

As described previously, the sum of the consultation times of previous patients consulted by the same physician could affect the waiting time of a patient. These consultation times would be influenced by the speed of the physician’s consultation because the distribution of consultation times could differ even among physicians who diagnose and treat the same disease in the same department. In addition, the interval between each patient’s consultations affects the waiting time. This interval is inevitable, but sometimes it could be long because of personal factors, such as the physician taking phone calls or attending to other tasks. Long intervals increase waiting times for subsequent patients and decrease the efficiency of the entire consultation service process. Therefore, we defined two features as the characteristics of a physician: the speed and efficiency of each physician’s consultation. Speed is the average consultation time of a physician within $\tau$ min prior to patient registration, whereas efficiency is the number of consulted patients within $\tau$ min prior to patient registration. In this study, we set $\tau$ as 60. Speed and efficiency have an inverse relationship only if the physician consulted without any break time. However, this situation is impossible in most cases due to inevitable intervals.

### Data preprocessing

After feature engineering, we constructed a dataset comprising nine observed variables and a target variable (i.e., waiting time). We divided the dataset into training and test sets at a ratio of 8:2 while maintaining a consistent ratio of instances per physician. Given that the waiting time showed a right-skewed distribution, we applied a square root transformation. The interquartile method was then used to remove outliers. Furthermore, we applied min-max normalization to the observed variables to prevent the effect of certain large-scale variables. Preprocessing was initially performed with the training dataset, and a threshold of the training dataset was applied to the test dataset. After preprocessing, 8,970 instances of the training dataset and 2,250 instances of the test dataset remained, and each was used for the training and evaluation of the ML models. Table 3 presents the statistics for each variable in the dataset.

### Prediction of waiting time for deriving an accurate relationship

We compared the prediction performance of different ML models to identify the model that best represented the relationship between waiting time and observed features. Linear models included LR and LR with regularization terms: elastic net (Elastic). Nonlinear models included support vector machines (SVM), RF [42], light gradient boosting machines (LightGBM) [43], extreme gradient boosting (XGBoost) [44], and multilayer perceptron (MLP). We also employed advanced neural network-based models to represent nonlinear relationships well. These models included TabNet [45] and FT-Transformer [46], which have recently demonstrated state-of-the-art prediction performance on several tabular datasets. For hyperparameter tuning, Bayesian optimization with a five-fold cross-validation was conducted. The R-squared ($R^2$), mean absolute error (MAE), and RMSE were computed for each model. The root mean square log error (RMSLE), which incurs larger penalties for underestimation, was also computed.

Table 4 presents a comparison of the ML models, with the average results on test datasets across five training runs using random seeds. LightGBM had the highest prediction accuracy, and it even had the lowest RMSLE. Notably, the LR model, which assumes linearity between service features and waiting times, showed the lowest accuracy compared with the other models that can represent nonlinear relationships. Given the interpretability of the model, the linear model was mainly used to understand and identify healthcare service operational situations. However, these results indicate that the assumption of linearity fails to accurately capture and represent their relationship, suggesting that a nonlinear assumption is necessary for more precise estimation. As a result, LightGBM was chosen as the model that best represented the relationship between the service features and waiting times. Conversely, the predictive performance of advanced neural network-based models, such as TabNet and FT-Transformer, fell short of the best results achieved by the other ML methods. This result suggests the inherent difficulty of the waiting time prediction problem due to the complex relationship between the features and waiting times, as reported in previous studies [7, 10, 17].

**Table 4:** Performance comparison of ML models

Model	RMSE	MAE	RMSLE	$R^{2}$
LR	16.327	11.910	0.520	0.496
Elastic	16.318	11.909	0.521	0.497
SVM	15.392	11.230	0.488	0.552
RF	15.115	11.034	0.480	0.568
LightGBM	**14.975**	**10.889**	**0.468**	**0.576**
XGBoost	15.029	10.921	0.471	0.573
MLP	15.467	11.225	0.492	0.562
TabNet	15.261	11.210	0.487	0.560
FT-Transformer	15.327	11.276	0.496	0.556

**Fig. 5:**
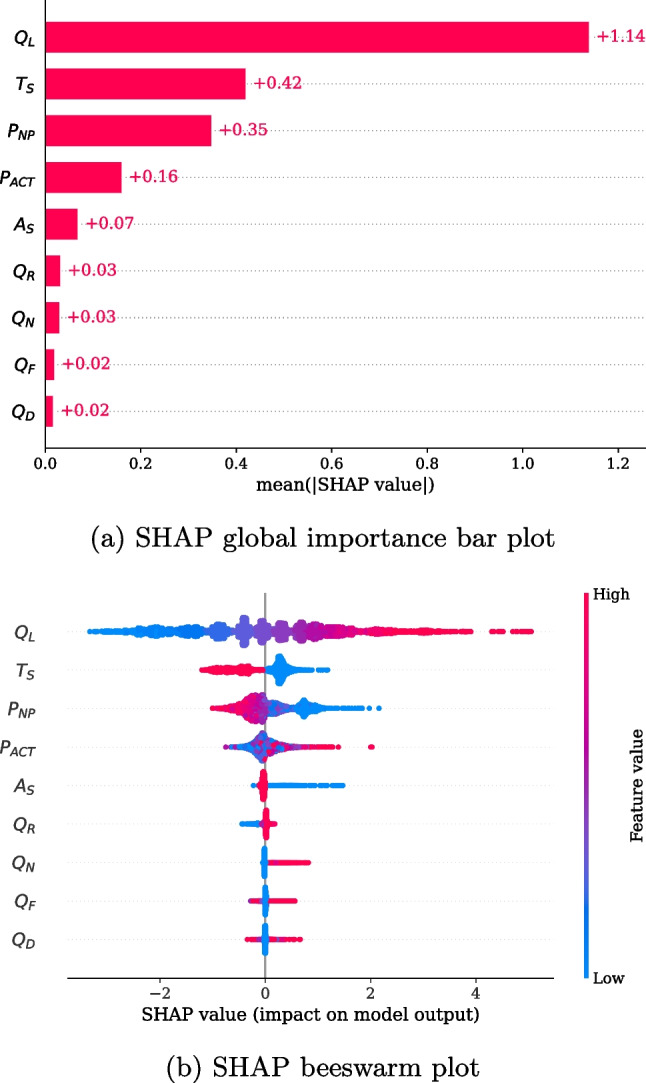
Feature importance at the global level

### Understanding the importance of features on waiting time

We used TreeSHAP [47] to interpret the relationship between features and waiting times because LightGBM, based on the tree model, was the best model in our experiments. Figure 5a shows the global importance of each feature that affected the prediction of waiting times based on the SHAP values. The influence of $Q_L$ was the most significant, followed by $T_S$ and $P_{NP}$. However, simple quantitative values could not determine whether these features have a positive or negative effect on waiting times. To identify the overall local importance according to the feature value, a SHAP beeswarm plot sorted by global importance is shown in Fig. 5b. Each point represents a SHAP value for each feature and is colored depending on the feature value. This plot also represents the distribution of values based on the line thickness. According to these results, a longer $Q_{L}$ results in higher SHAP values, which corresponds to an increase in the predicted waiting times. Through correlation analysis, we found that $Q_L$ and its corresponding SHAP values had a strong linear relationship ($r = .972, p < .0001$)^2^. By contrast, other features belonging to the queue factor had a relatively low influence on waiting time. $Q_N$, $Q_F$, and $Q_D$ were distributed at low values and had relatively less influence on decreasing the waiting time. However, waiting times may increase when these values are high. Particularly, $Q_N$ exhibited the most consistent tendency among the three. Interestingly, this result was consistent with the nurses’ statements that waiting time could increase when patients visiting the hospital for the first time or being consulted in a new department accounted for a large percentage of the queue. When $A_S$ was 1, its importance values were negative and close to 0, and when $A_S$ was 0, its importance values were mostly positive. Moreover, if the patient registration time was within the smooth flow time zone (i.e., $T_S=1$), the SHAP values were negative. In the opposite case (i.e., $T_S=0$), the SHAP values were positive and close to 0. Registration in the smooth flow time zone significantly affected the reduction of waiting times, but in the opposite case, it had a relatively insignificant effect. Finally, $P_{NP}$ had a significantly negative or positive effect on waiting times when the value was high or low, respectively. Alternatively, $P_{ACT}$ had less influence than $P_{NP}$ but exhibited positive or negative importance as it increased or decreased, respectively.

**Fig. 6:**
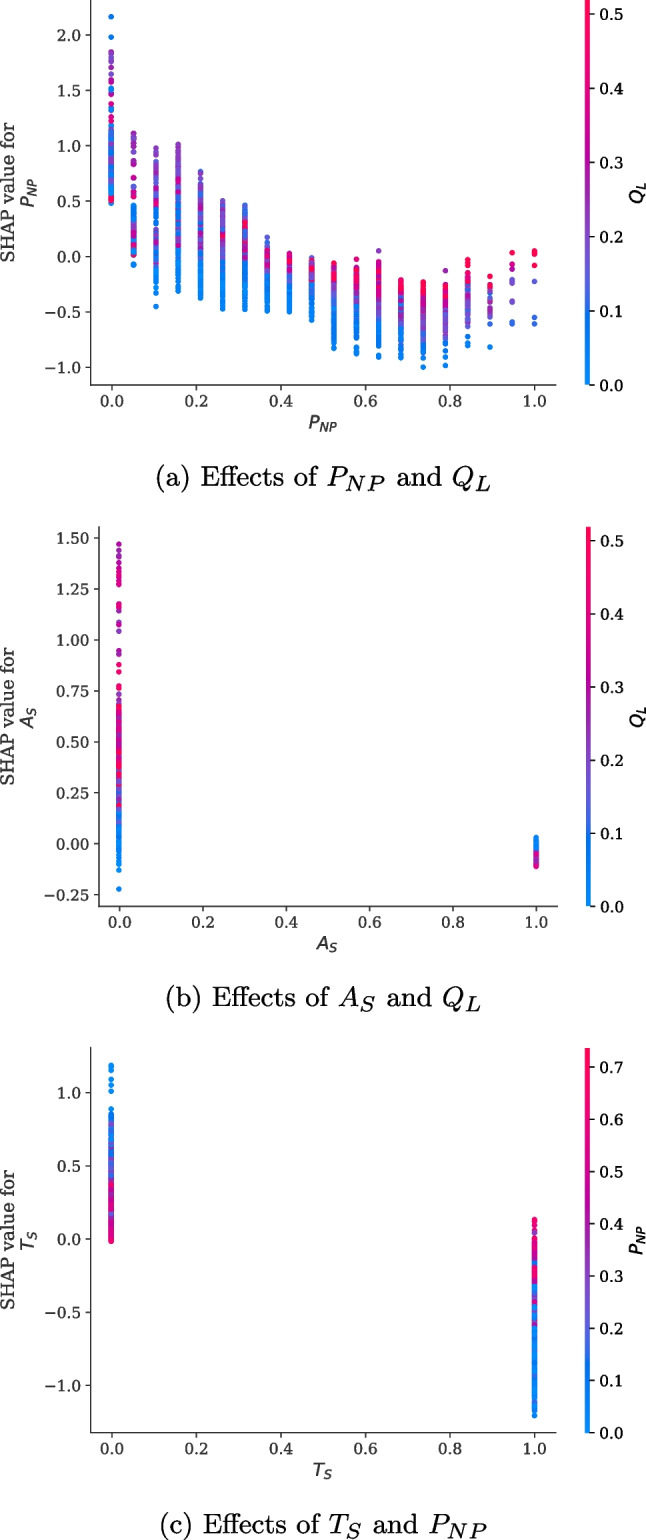
Examples of feature dependency

To analyze the effects of single features on the prediction of waiting times and their interaction with other features, feature dependency plots are presented in Fig. 6. The x- and y-axes represent the values of the feature and its corresponding SHAP values, respectively, and the colors represent the values of a subject feature. We conducted this analysis for combinations of all features. As a result, the three combinations showed distinct relationships with the largest interaction effect. Figure 6a presents the effects of $P_{NP}$ and $Q_L$ on prediction. As shown in the figure, $P_{NP}$ had a negative correlation with its corresponding SHAP values ($r = -.880, p < .0001$), and it had a negative effect on waiting times when its value exceeded 0.421 (8.0 patients). Moreover, the SHAP values for $P_{NP}$ with higher $Q_L$ values were higher, regardless of the $P_{NP}$ values. This result suggests that the effect of $P_{NP}$ on decreasing waiting time was offset by the increase in $Q_L$. Figure 6b shows the effects of $A_S$ and $Q_L$ on the model output. For most patients who visited with appointments, the SHAP value for $A_S$ was unaffected, whereas variations in the SHAP value were observed when patients visited without an appointment. Interestingly, this variation tended to increase with $Q_L$. Finally, we explored the effects of $T_{S}$ and $P_{NP}$. As shown in Fig. 6c, as $P_{NP}$ increased, the SHAP value for $T_S$ became close to 0; that is, $P_{NP}$ tended to offset the overall effect of $T_{S}$ on the predicted waiting times.

### Determining the model for waiting time prediction service

We compared the performance of the model trained using asymmetric loss functions. For this, we selected LightGBM, which showed the best performance in the above experiment, as a base model. The other ML models trained using asymmetric loss functions could also be utilized for prediction after performance comparisons among the models. The candidates of $\alpha$ for Quad-Quad and $\beta$ for LINEX were set as $\{$0.5, 0.6, 0.7, 0.8, 0.9, 1.0$\}$^3^ and $\{0.1, 0.2, \dots , 0.9, 1.0\}$, respectively. The other hyperparameter tuning settings were the same as those in Section 3.4. Here, we introduced DAES as a score function to identify the optimal set of hyperparameters, including the selection of an asymmetric loss function. DAES evaluates a trained model’s performance by considering patient dissatisfaction resulting from underestimation and excessive overestimation. We set the $\gamma$ of DAES to 30 min, considering the nurses’ concerns after the interview, and $e(\cdot )$ as the RMSE. In this study, five-fold cross-validation was used to improve reliability with respect to sampling variation. To confirm the robustness of the model’s performance with respect to hyperparameters, we performed five iterations of cross-validation with random seeds and reported the average results in Table 5. According to the results, the ratio of underestimation $\rho _{(\epsilon > 0)}$ gradually decreased and RMSE increased as the values of parameters $\alpha$ and $\beta$ increased. Specifically, when a model was trained using Quad-Quad with $\alpha =1.0$, its accuracy was significantly reduced, although it showed the lowest ratio of underestimation. Although a model trained using LINEX with $\beta =0.1$ achieved high accuracy, it could not prevent underestimation. This finding implies the existence of a trade-off between underestimation and accuracy. On the basis of the DAES results, these models were not notably evaluated for their performance. Conversely, a model trained by Quad-Quad with $\alpha =0.8$ outperformed the other models in terms of DAES, indicating that it had a proper balance between underestimation and accuracy. These results demonstrate that the DAES allowed for the evaluation of goodness of fit by considering the trade-off.

**Table 5:** Cross-validation results with different loss functions

Loss			RMSE	$\rho _{(\epsilon > 0)}$	RMSE($\epsilon > 0$)	$\rho _{(\epsilon < 0)}$	RMSE($\epsilon < 0$)	DAES(30)
Quad-Quad	$\alpha$	0.5	15.656	0.466	18.337	**0.534**	**12.102**	9.204
		0.6	15.708	0.407	17.949	0.593	13.247	8.150
		0.7	16.075	0.353	17.425	0.647	14.668	7.292
		0.8	17.145	0.290	16.895	0.710	16.724	**6.729**
		0.9	19.911	0.196	16.371	0.804	20.298	7.094
		1.0	65.898	**0.006**	**7.232**	0.994	66.072	63.694
LINEX	$\beta$	0.1	**15.626**	0.442	18.261	0.558	12.390	8.776
		0.2	15.658	0.423	17.990	0.577	12.951	8.414
		0.3	15.744	0.402	17.881	0.598	13.371	8.031
		0.4	15.887	0.376	17.682	0.624	13.952	7.572
		0.5	16.181	0.355	17.649	0.645	14.579	7.277
		0.6	16.517	0.338	17.514	0.662	15.277	7.115
		0.7	16.893	0.314	17.351	0.686	15.995	6.825
		0.8	17.507	0.290	17.330	0.710	16.883	6.730
		0.9	17.983	0.279	17.266	0.721	17.546	6.833
		1.0	19.185	0.240	17.296	0.760	19.062	7.025

**Fig. 7:**
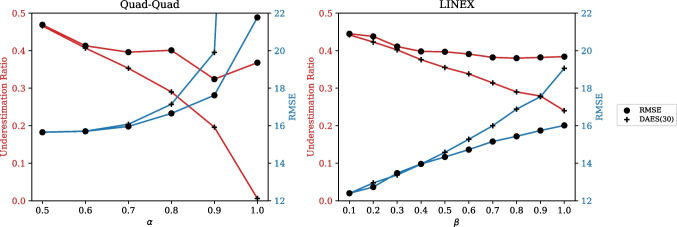
Comparative results across the degrees of asymmetry by score functions

In general, RMSE is utilized as a score function for prediction models, with a primary emphasis on accuracy. To ascertain the effectiveness of DAES as a score function for waiting time prediction considering dissatisfaction, we conducted a comparative analysis of the performances of the best model determined by DAES and RMSE across the degrees of asymmetry. The comparative results are graphically presented in Fig. 7. In both score functions, an increase in the degree of asymmetry consistently reduced the underestimation ratio, accompanied by an increase in RMSE (i.e., a decrease in accuracy). This observation reaffirms the inherent trade-off between underestimation and accuracy. Notably, in the case of DAES, the reduction in the underestimation ratio was more pronounced compared with the case of RMSE. Given that RMSE primarily emphasizes accuracy without considering the intention of underestimation, it may not be the most suitable choice for capturing this aspect. By contrast, DAES demonstrates its effectiveness in preventing underestimation. In other words, utilizing DAES as a scoring function facilitates the identification of proper models that can incorporate preventing underestimation as a desirable attribute.

**Table 6:** Evaluation results of the best models determined by the parameters of DAES

Score	Loss	RMSE	$\rho _{(\epsilon > 0)}$	RMSE($\epsilon > 0$)	RMSE($\epsilon < 0$)	$\rho _{(\epsilon < -\gamma )}$						
						$\gamma =0$	$\gamma =10$	$\gamma =20$	$\gamma =30$	$\gamma =40$	$\gamma =50$	$\gamma =60$
RMSE	MSE	14.989	0.459	18.298	11.452	0.541	0.185	0.042	0.010	0.002	0	0
	LINEX(0.1)	14.939	0.437	18.066	11.963	**0.563**	0.209	0.047	0.012	0.002	0	0
DAES(10)	MSE	14.949	0.460	18.084	11.636	0.540	0.189	0.042	0.011	0.003	0	0
	LINEX(0.1)	14.918	0.436	17.922	12.090	0.564	**0.207**	0.049	0.014	0.003	0	0
DAES(20)	MSE	14.987	0.460	18.189	11.574	0.540	0.190	0.041	0.011	0.002	0	0
	LINEX(0.4)	15.201	0.365	17.701	13.555	0.635	0.303	**0.073**	0.021	0.004	0	0
DAES(30)	MSE	14.993	0.459	18.221	11.571	0.541	0.189	0.042	0.010	0.003	0	0
	Quad-Quad(0.8)	16.476	0.271	16.776	16.362	0.729	0.429	0.146	**0.045**	0.011	0.003	0
DAES(40)	MSE	14.947	0.461	18.068	11.635	0.539	0.190	0.042	0.010	0.002	0	0
	Quad-Quad(0.9)	19.475	**0.173**	16.857	19.977	0.827	0.607	0.287	0.088	**0.027**	0.006	0.001
DAES(50)	MSE	14.991	0.461	18.178	11.585	0.539	0.188	0.042	0.010	0.002	0	0
	Quad-Quad(0.9)	19.407	0.181	16.423	20.010	0.819	0.589	0.277	0.090	0.029	0.008	0.001
DAES(60)	MSE	14.938	0.463	17.872	11.845	0.537	0.190	0.046	0.010	0.002	0	0
	Quad-Quad(0.9)	19.514	0.177	16.569	20.091	0.823	0.600	0.285	0.090	0.030	0.007	0.001

**Fig. 8:**
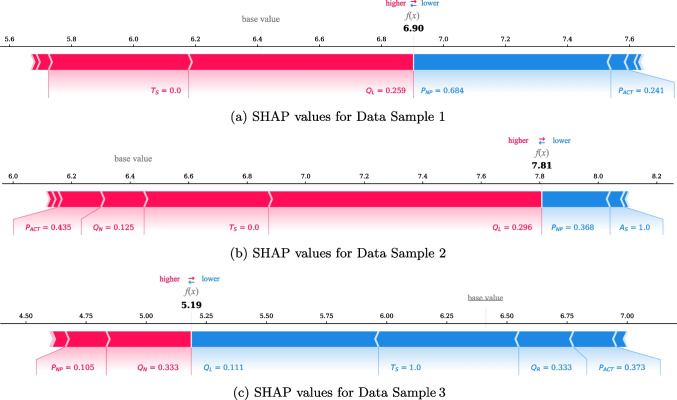
Examples of feature importance at the local level

DAES penalizes not only underestimation but also excessive overestimation. The penalty for excessive overestimation is applied only when it exceeds a predefined time threshold $\gamma$, which practitioners can adjust as a parameter. To confirm the ability of DAES to control excessive overestimation, we evaluated the best models on the test datasets. These best models were trained with asymmetric loss function and tuned using different DAES parameters. The candidate value for $\gamma$ was set as {10, 20, 30, 40, 50, 60}. We also evaluated model performance trained with MSE across DAES parameter settings to examine the compatibility between score and loss functions. The results are reported in Table 6. As the time threshold $\gamma$ increased, a loss function with greater asymmetry was selected, leading to a decrease in the underestimation rate and a shift in the model’s prediction distribution toward overestimation. In other words, by adjusting the permissible range for overestimation errors, the prediction distribution of the model could be deterred from leaning considerably toward overestimation. Hence, with the parameter $\gamma$, practitioners can control the degree of the excessive model’s overestimation. However, when $\gamma$ exceeded 40 min, no further changes in the prediction distribution based on the parameter were observed. The reason is that the time threshold $\gamma$ exceeded the model’s prediction error range, and the penalty for overestimation became negligible. Therefore, practitioners should consider the model’s prediction range when determining the value of $\gamma$. Furthermore, when employing MSE, which is a symmetric loss function, the performance remained consistent regardless of the DAES parameter variations. This result suggests utilizing DAES and an asymmetric loss function concurrently can be effective for a predictive model considering outpatient dissatisfaction.

### Providing the expected waiting time and its explanation

SHAP values explain how the predicted waiting times were affected by each feature. Thus, we used TreeSHAP to interpret the LightGBM trained with Quad-Quad with $\alpha =0.8$, which showed the best performance in terms of DAES(30). Figure 8 shows examples of the feature importance at the local level. The root-squared waiting time is presented in colored plots. Red and blue represent features that increase or decrease the prediction value, respectively. The length of each feature corresponds to the extent of its effect on the SHAP value. As shown in Fig. 8a, the model predicted an actual value of 5.88 (34.52 min) to 6.90 (47.61 min). The most important feature in this case was $P_{NP}$, with a value of 0.684 (13 patients), which decreased the prediction value. Moreover, the $P_{ACT}$ value of 0.241 (approximately 3.6 min) decreased the waiting times. By contrast, $Q_L$ and $T_S$ with values of 0.259 (7 patients) and 0, respectively, contributed to an increase in waiting times. Therefore, physicians’ high efficiency and short consultation time reduced the expected waiting times despite the long queue lengths and visiting a nonsmooth flow time zone ($T_S=0$). As shown in Fig. 8b, the predicted value was 7.81, corresponding to 61.00 min. The actual waiting time for this instance was 53.52 min. $Q_L$ with a value of 0.296 (8 patients) had the greatest effect on increasing the prediction. In view of the nonsmooth flow time zone, a newly visiting patient in the queue (i.e., $Q_N=0.125$; one patient) and long consultation times of assigned physicians (i.e., $P_{ACT}=0.435$; 6.48 min) affected the increase. Although $P_{NP}$ contributed to decreasing the prediction value, the effect was somewhat insignificant because its value (0.368, 7 patients per hour) was relatively small compared with the result in Fig. 8a. Lastly, Fig. 8c shows the result of the model that predicted a value of 5.19 corresponding to a value of 26.94 min, and its actual waiting time was 26.18 min. $Q_L$ and $T_S$ with values of 0.111 (3 patients) and 1, respectively, contributed to a decrease in waiting times. In the queue, newly visiting and returning patients had an equal ratio (i.e., $Q_{N}=Q_{R}=0.333$). However, the importance of the newly visiting patient ratio on waiting time was marginally larger than that of the revisiting patient ratio. Specifically, although the ratio of newly visiting patients contributed to an increase in waiting time, the ratio of returning patients had the opposite effect, contributing to a decrease in waiting times. Conversely, the variables related to the physician factor had similar absolute values of effect on waiting time. However, the physician’s slightly fast consultation speed (i.e., $P_{ACT}$=0.373; 5.56 min) contributed to a decrease in waiting time, whereas low consultation efficiency (i.e., $P_{NP}$=0.105; 2 patients per hour) led to an increase in waiting time.

**Table 7:** Performance comparison of ML models for the NS department

Model	RMSE	MAE	RMSLE	$R^{2}$
LR	14.772	11.126	0.662	0.439
Elastic	13.892	10.553	0.642	0.477
SVM	13.015	9.666	0.594	0.551
RF	13.007	9.632	0.601	0.541
LightGBM	**12.729**	**9.494**	**0.595**	**0.561**
XGBoost	12.796	9.506	0.597	0.556
MLP	13.114	9.795	0.605	0.526
TabNet	13.450	9.986	0.626	0.510
FT-Transformer	13.286	9.821	0.598	0.521

Providing this type of information to nurses will enable them to inform the expected waiting time and explain the reasons to their patients. For example, a nurse can provide the following information to a patient: "Your waiting time could have been longer due to 13 patients currently waiting and registration in a busy time zone, but your doctor has been consulting quickly for the last hour, so your expected waiting time is about 50 minutes", or "Your waiting time is predicted to be about 27 minutes. Although there are only three patients ahead of you, the increase in waiting time is expected because a newly visiting patient is in the queue. Also, your attending physician is conducting consultations quickly, but it seems to be slightly delayed due to unforeseen personal reasons. So, a slight increase in waiting time is expected". With this, we expect patients to understand the factors that they could not observe in the waiting room and their predicted waiting time.

**Fig. 9:**
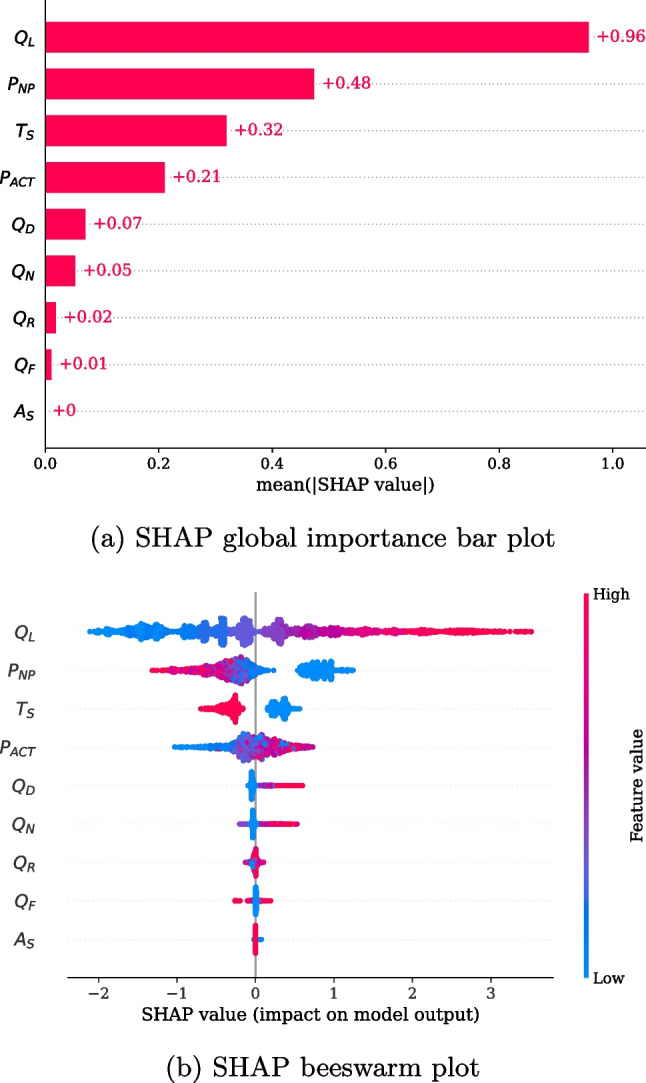
Feature importance at the global level for the NS department

**Fig. 10:**
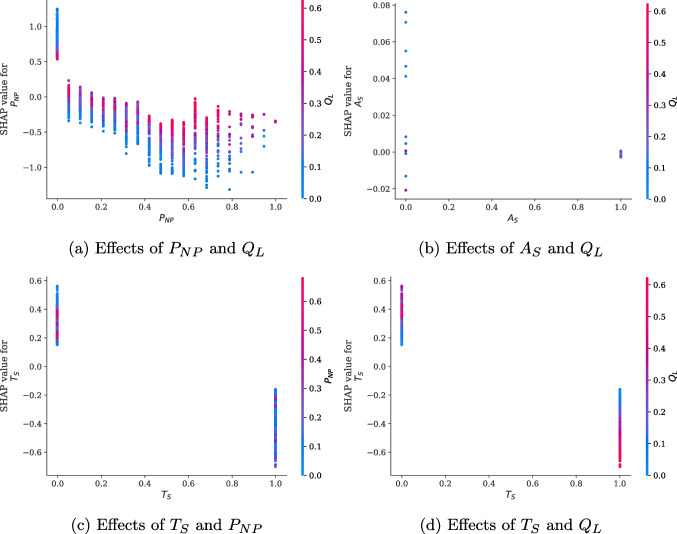
Examples of feature dependency for the NS department

### Additional case study on the NS department

We applied our framework to the NS department of the same hospital to validate the applicability of our framework and compare analysis results between different departments. For the NS department, the mean waiting time was 29.31 min with a standard deviation of 20.55 min, whereas the mean and standard deviation of consultation time were 5.48 min and 3.31 min, respectively. We extracted event logs from the HIS for the same period. After discussion with medical workers and observations of patient flow, we set parameters for data construction, specifically $\tau$ was set to 60 and periods for $T_{S}$ were set as the start time of each consultation (i.e., between 08:00 and 10:00 and between 13:00 and 15:00). After data preprocessing, 3,773 data instances from 2,741 patients were utilized to train and evaluate the ML models.

The result of the performance comparison is presented in Table 7. The LightGBM exhibited superior performance even in the NS department. Accordingly, To understand the importance of features on waiting time in the NS department, we applied TreeSHAP to the LightGBM and displayed the bar plot and beeswarm plot in Fig. 9. As shown in Fig. 9a, the number of queues had the most significant effect on waiting times, aligning with the result of the EM department. In the NS department, a doctor’s consultation efficiency had a greater influence on waiting times than the that on the smooth flow time zone. Notably, patients’ appointment status almost had no effect on waiting times. In addition, the effects of the newly visiting patient ratio and department first-time outpatient ratio were more pronounced among the waiting queue factor compared with the EM department. These analysis results from these departments suggest that the importance of each feature related to waiting time can differ based on the unique clinical settings of each medical department. Furthermore, as illustrated in Fig. 9b, the trend of changes in the SHAP values for each feature value in the NS department aligned with the EM department’s analysis results. These consistent results confirm the applicability of our analytical approach using IML.

Figure 10 displays the feature dependency plots specific to the NS department. As illustrated in Fig. 10a, the relationship between SHAP values for $P_{NP}$ and $Q_{L}$ was similar to the patterns observed in the EM department. However, different from the EM department, the effect of $P_{NP}$ decreased as $Q_{L}$ increased when $P_{NP}$ was equal to 0. Thus, in the NS department, the increase in $Q_{L}$ offset the effect of $P_{NP}$ on waiting times. Figure 10b and c represent the dependency plots of combinations that showed distinct relationships in the EM department. However, these relationships were absent in the NS department. Conversely, in NS department, a different relationship was observed, which was not found in the EM department. Figure 10d reveals that as $Q_{L}$ increased, the effect of $T_{S}$ on waiting times became more pronounced. Hence, when $Q_{L}$ was short, the patient registration time within $T_{S}$ did not significantly affect the waiting time; however, when $Q_{L}$ was long, the patient registration time within $T_{S}$ influenced the reduction in waiting times more.

**Table 8:** Evaluation results of the best models determined by the parameters of DAES for the NS department

Score	Loss	RMSE	$\rho _{(\epsilon > 0)}$	RMSE($\epsilon > 0$)	RMSE($\epsilon < 0$)	$\rho _{(\epsilon < -\gamma )}$						
						$\gamma =0$	$\gamma =10$	$\gamma =20$	$\gamma =30$	$\gamma =40$	$\gamma =50$	$\gamma =60$
RMSE	MSE	12.694	0.500	14.632	10.405	0.500	0.139	0.027	0.004	0.002	0.001	0.001
	Quad-Quad(0.6)	12.613	0.447	14.083	11.274	0.553	0.190	0.038	0.005	0.003	0.001	0.001
DAES(10)	Quad-Quad(0.6)	12.606	0.439	14.087	11.314	0.561	0.194	0.037	0.004	0.003	0.001	0.001
DAES(15)	Quad-Quad(0.7)	12.966	0.363	13.810	12.459	0.637	0.274	0.057	0.009	0.003	0.002	0.001
DAES(20)	Quad-Quad(0.8)	13.794	0.293	13.274	14.000	0.707	0.362	0.086	0.021	0.003	0.002	0.001
DAES(25)	Quad-Quad(0.9)	15.603	0.204	12.824	16.230	0.796	0.487	0.153	0.040	0.008	0.003	0.001
DAES(30)	Quad-Quad(0.9)	15.906	0.202	12.735	16.619	0.798	0.504	0.163	0.046	0.008	0.003	0.001
DAES(40)	Quad-Quad(0.9)	16.075	0.193	12.976	16.732	0.807	0.526	0.173	0.041	0.008	0.003	0.001
DAES(50)	Quad-Quad(0.9)	15.819	0.202	12.675	16.524	0.798	0.500	0.161	0.043	0.008	0.003	0.001
DAES(60)	Quad-Quad(0.9)	16.061	0.190	13.013	16.697	0.810	0.526	0.176	0.041	0.008	0.003	0.001

To identify the most suitable model for waiting time prediction service in the NS department considering patient dissatisfaction, we applied our approach using asymmetric loss function and DAES to train the model and identify the optimal combination of hyperparameters. Table 8 shows the evaluation results of models trained with asymmetric loss function across different parameters of DAES. Given that the waiting times in the NS department were relatively shorter than those in the EM department, we added {15, 25} to candidates of $\gamma$. The results showed that the loss function with pronounced asymmetry was chosen, as the time threshold $\gamma$ increased, thereby decreasing the model’s underestimation ratio and increasing its overestimation ratio. This trend aligned with the results from the EM department. For the NS department, models with $\gamma$ values beyond 25 min showed no further changes in the prediction distribution as $\gamma$ values. Thus, practitioners of the NS department in this hospital should set an appropriate DAES within 25 min.

## Concluding remarks

In practical settings, the use of IML and an asymmetric loss function for outpatient waiting time prediction offers several advantages. First, the use of IML enabled us to identify the effects of different service operational features on waiting times. The quantitative value output from IML can provide valuable insights for decision makers to understand the underlying features of hospital systems and make informed improvements to reduce waiting times. In practice, on the basis of the analysis conducted with IML, we recommended the EM department to encourage patients to make appointments. We also suggested an effective allocation of physicians during a smooth flow time zone and an increase in the number of medical workers. For the NS department, we recommended inducing newly visiting and first-visiting patients to schedule their consultation time into a smooth flow time zone and effectively assigning physicians considering their consultation style. Second, ML-based waiting time prediction models must provide predictions that align with patients’ perception and satisfaction. This study addressed this requirement by developing a method that incorporates asymmetric loss functions and DAES to predict dissatisfaction-aware waiting times. The use of asymmetric loss functions enabled the model not to underestimate the expected waiting times, which, in turn, reduced the likelihood of patients experiencing dissatisfaction due to unexpected short waiting times. In addition, the use of DAES ensures the model to make accurate predictions with suitable asymmetry, reducing the risk of excessive overestimation inherent in using asymmetric loss functions. It also provides practitioners the flexibility to adjust DAES parameters, allowing for tailored asymmetry in consideration of outpatient dissatisfaction.

To the best of our knowledge, this study is the first attempt to use IML to accurately estimate the extent to which focal operational features affect outpatient waiting times. The majority of previous studies that aim to understand operational situations and identify service improvements in healthcare were limited in accurately capturing the complex relationship between features and waiting times, primarily due to linearity assumption. Our study empirically reveals the necessity of considering a nonlinear relationship between waiting times and operational features in outpatient care services. By employing IML, we overcome the limitations of previous research, enabling a more precise estimation and interpretation of nonlinear relationships. Thus, our study contributes to filling this research gap that has not yet been addressed in healthcare service management. Moreover, this study represents a novel approach to employing asymmetric loss function and DAES for training of waiting time prediction model, specifically aimed at reducing both underestimation and excessive overestimation. Thus, our study also contributes to bridging the research gap by adapting patient-centered philosophies, which have been previously overlooked in the literature on outpatient waiting time prediction.

The methodological contribution and practical applicability of this study were validated through a case study on the importance estimation of operational features affecting the waiting times in the EM and NS departments’ outpatient services of one of the largest hospitals in South Korea. To highlight the practical contributions of our framework and provide concrete examples of its application, we present several valuable applications in the healthcare setting as follows: (Real-time patient notifications) One key application is the provision of real-time patient notifications. For instance, many patients have expressed frustration with not knowing how long they may need to wait for their consultations. Our case studies demonstrate that our methodology accurately predicts waiting times in 1-min units, providing a higher level of granularity compared with traditional 15- and 30-min units. Our approach also offers explanations for its prediction. Providing patients with precise updates about their consultation can enhance the overall patient experience, reducing anxiety and dissatisfaction. (Operations improvement) From the perspective of outpatient operations, our framework allows healthcare providers to better understand the factors contributing to longer waiting times. On the basis of the advantages of IML discussed earlier, we can identify the specific reasons behind extended waiting times. This knowledge allows for strategic interventions, such as adding extra nurses, to reduce waiting times or adjusting appointment schedules. The ability to determine the causes of longer waiting times is a significant contribution to operational efficiency. (Minimizing patient dissatisfaction) Our research emphasizes the importance of minimizing potential patient dissatisfaction due to inaccurate predictions. By incorporating an asymmetric loss function and DAES into our model, we can reduce the effects of inaccurate predictions on patients. This not only enhances the patient experience but also reduces the risk of dissatisfaction, which can negatively affect the reputation of healthcare service providers. In conclusion, we believe the proposed framework will serve as a valuable tool for monitoring, managing, and enhancing hospital outpatient services.

## Data Availability

Data are not publicly available due to security, privacy, or ethical restrictions.

## Footnotes

1 In Fig. 4, the black line represents the mean waiting time, and the gray shade area represents the 95% confidence interval.

2 *r*: Pearson’s correlation coefficient, *p*: p-value.

3 In case of $\alpha =0.5$, the Quad-Quad performs similarly to MSE. To compare the difference in the degree of asymmetry, we added $\alpha =0.5$ in the candidates.
